# Effective Interventions for Reducing the Negative Effects of Night Shifts on Doctors’ and Nurses’ Health and Well-Being: A Systematic Review

**DOI:** 10.7759/cureus.83385

**Published:** 2025-05-03

**Authors:** Michael Shakhloul, Ahmed Amer, Mina Zekry, Mohamed Elgewely, Abanoub Saleeb, Shenoda Ghobrial, Mario Zaghar A Shehata, Ibrahim Abouelkhir, Muhammad O Kamal, Bishoy Manqaryos, Mahmoud Abdulfattah, Ahmed Syed, David Shakhloul, Mina Kamel

**Affiliations:** 1 Emergency Medicine, Royal Surrey County Hospital, Guildford, GBR; 2 Emergency, Royal Surrey County Hospital, Guildford, GBR; 3 Trauma and Orthopaedics, East Kent Hospitals University National Health Service (NHS) Foundation Trust, Margate, GBR; 4 General Surgery, Guy's and St Thomas' National Health Service (NHS) Foundation Trust, London, GBR; 5 Internal Medicine, National Health Service (NHS), Gloucester, GBR; 6 Adult Critical Care, St George's University Hospitals National Health Service (NHS) Foundation Trust, London, GBR; 7 Emergency Medicine, Frimley Park Hospital, Frimley, GBR; 8 Orthopaedics, Suzan Mubarak Hospital, Minya, EGY; 9 Emergency, National Health Service (NHS), London, GBR

**Keywords:** healthcare work force, night shift, occupational health doctor, shift work sleep disorders (swsd), shift work tolerance, sleep problems

## Abstract

Working night shifts is a reality for many doctors and nurses, but it often comes at a cost to their sleep, alertness, and overall well-being. This systematic review looked at a wide range of strategies tested to ease these effects and support those working through the night. Across 74 studies, we explored interventions including bright light exposure, melatonin, dietary changes, scheduled naps, medications like modafinil and caffeine, and complementary approaches such as aromatherapy, exercise, and acupuncture. Bright and blue-enriched light, melatonin, modafinil, and napping stood out as consistently helpful in boosting alertness and sleep quality. Other interventions showed promise in certain settings, but were less consistent overall. These findings suggest that no single solution fits everyone, and that a personalized, flexible approach combining different strategies may work best. There’s a clear need for more long-term, real-world studies to help healthcare professionals stay well while caring for others overnight.

## Introduction and background

Over the past few decades, a variety of interventions have been explored in an attempt to reduce the negative effects of night shifts on healthcare workers [1-74]. These interventions range from pharmacological treatments (such as melatonin and stimulants) to behavioral approaches (such as light therapy, diet modifications, and napping strategies). However, despite the numerous studies conducted on this topic, there remains a lack of consensus regarding the most effective interventions, as well as their optimal application in different settings.

Night shift work is a prevalent and necessary component of healthcare services, especially for doctors and nurses who are integral to patient care in hospitals and other healthcare settings. However, working night shifts can have significant negative effects on the health and well-being of healthcare professionals. The disruption of the natural circadian rhythm, which governs sleep-wake cycles, is the primary cause of these adverse effects [75]. As a result, night shift workers often experience sleep disturbances, increased fatigue, reduced cognitive function, mood disturbances, and other physiological consequences.

For doctors and nurses, the consequences of disrupted sleep and circadian misalignment are particularly concerning, as they can directly impact both their personal health and the quality of care they provide to patients. Cognitive impairments, such as decreased alertness and slower decision-making, can lead to errors in clinical judgment, which may jeopardize patient safety [76]. Additionally, prolonged exposure to night shift work has been linked to a higher risk of developing chronic conditions, including cardiovascular disease, obesity, diabetes, and gastrointestinal disorders [77].

This systematic review aims to provide a comprehensive evaluation of the interventions that have been tested to mitigate the negative effects of night shift work on the health and well-being of doctors and nurses. By synthesizing the findings from a variety of studies, this review seeks to identify the most effective strategies for improving the health and performance of healthcare professionals working night shifts.

## Review

Methods

This systematic review was conducted in accordance with a predefined protocol registered in the PROSPERO database (registration ID: CRD420251015466), ensuring methodological transparency and minimizing the risk of bias throughout the review process.

Studies were eligible for inclusion if they evaluated interventions specifically designed to reduce the negative effects of night shifts on the health and well-being of doctors and nurses. Exclusion criteria included studies that: (1) discussed the harms of night shift work without evaluating an intervention; (2) assessed multiple interventions within the same group without clear separation; (3) involved mobile applications or educational tools; and (4) focused solely on non-healthcare populations.

A comprehensive literature search was conducted using the PubMed database with the search term “night shift,” which captured studies containing the words “night,” “shift,” and the phrase “night shift.” The search was limited to articles published in English, with no date restrictions, and was last updated on March 3, 2025. Since only PubMed was searched, no duplicate records were identified.

**Figure 1:**
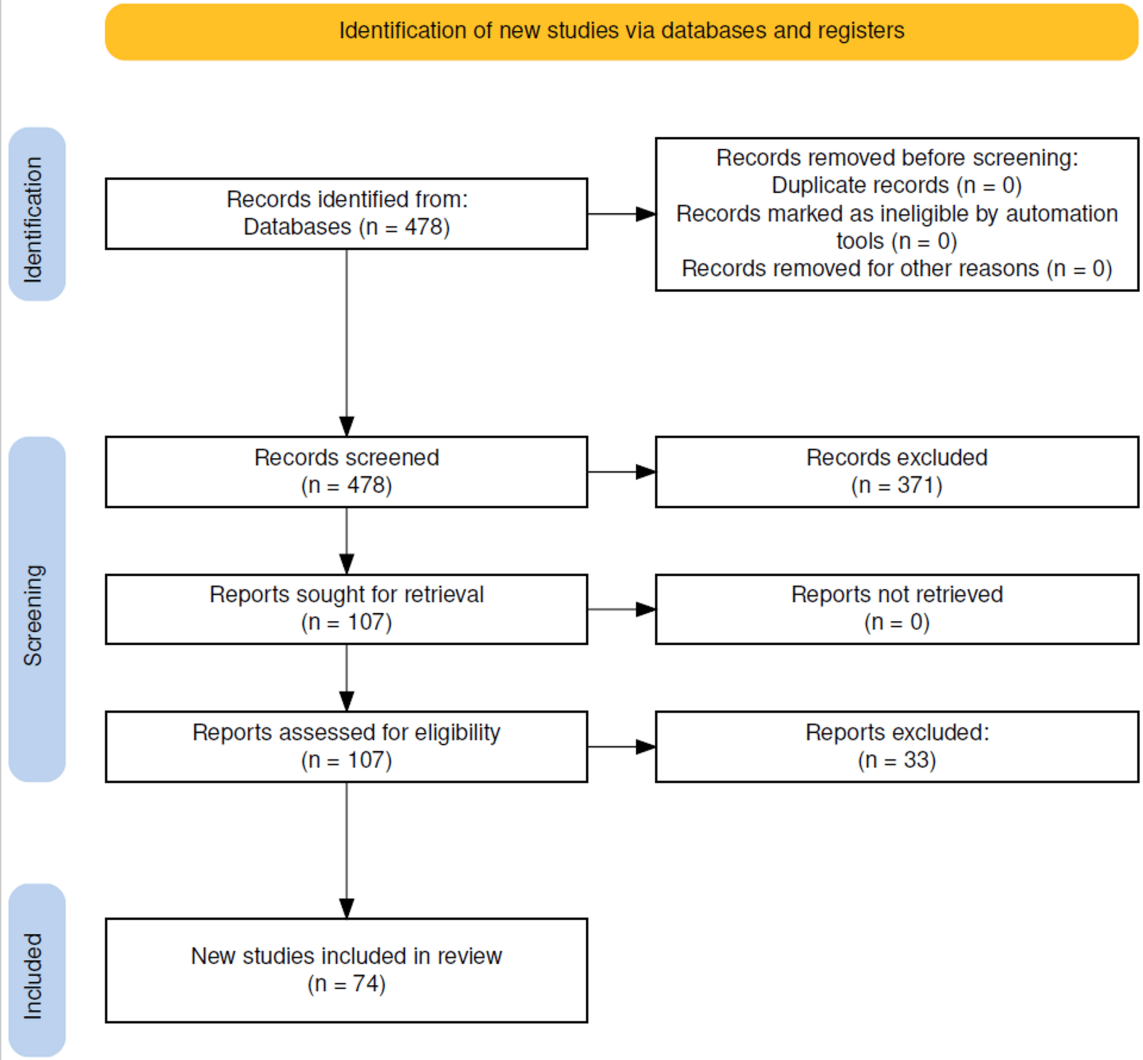
PRISMA flow diagram PRISMA 2020 flow diagram illustrating the study selection process. Image credit: [78]

The search initially yielded 478 studies. Titles and abstracts were screened for relevance, and 107 studies were identified as potentially eligible. Following full-text review and the application of the inclusion and exclusion criteria, 74 studies were included in the final analysis. The process of study selection is outlined in the PRISMA flow diagram (Figure 1).

Data were extracted from each study, including information on the type of intervention, study design, sample size, outcomes measured, and key findings. The studies were categorized by the primary intervention evaluated, such as light exposure, melatonin, dietary changes, napping, pharmacological agents, aromatherapy, physical exercise, and acupuncture.

Risk of bias was assessed using the Cochrane Risk of Bias 2 (RoB 2) tool, with adaptations made for crossover and non-randomized study designs. Each study was rated as having low risk, some concerns, or high risk of bias across five standard domains: the randomization process, deviations from intended interventions, missing outcome data, measurement of outcomes, and selection of the reported result. A summary of these assessments is presented in Table 1.

**Table 1:** Included studies

Study	Design	Sample Size	Intervention	Positive Effect	Risk of Bias	Justification of Risk of Bias Judgment
Bjorvatn et al. (2021) [1]	A placebo-controlled crossover study	35	Light	N	Some concerns	Bright light intervention is difficult to blind; Subjective outcomes.
Bjorvatn et al. (2007) [2]	A randomized controlled study	17	Light/melatonin	Y	Some concerns	Small sample size; Bright light intervention is difficult to blind; Subjective outcomes.
Lammers-van der Holst et al. (2021) [3]	A randomized controlled trial	29	Light	Y	Some concerns	Small sample size; Bright light intervention is difficult to blind; Subjective outcomes could be biased if participants guessed the treatment.
Cyr et al. (2023) [4]	A randomized controlled trial	57	Light	Y	Some concerns	Light intervention is difficult to blind. Subjective outcomes could be biased if participants guessed treatment.
Sunde et al. (2022) [5]	A counterbalanced crossover study	34	Light	Y	Some concerns	Crossover design is sensitive to carryover; Light intervention is difficult to blind.
Weisgerber et al. (2017) [6]	A counterbalanced crossover trial	19	Light	Y	Some concerns	Crossover design is sensitive to carryover; Light intervention is difficult to blind.
Horowitz et al. (2001) [7]	A randomized controlled trial	54	Light	N	Low risk	The only concern is the possibility of unblinding due to the nature of the intervention.
Huang et al. (2013) [8]	A randomized controlled study	82	Light	Y	Some concerns	Light intervention is difficult to blind; Primary outcomes (insomnia, anxiety, depression) are subjective.
Dawson et al. (1995) [9]	A randomized controlled study	36	Light/melatonin	Y	Low risk	The only concern is the possibility of unblinding due to the nature of the intervention.
Tanaka et al. (2011) [10]	A randomized crossover study	61	Light	Y	Some concerns	Crossover design is sensitive to carryover; Not blinded.
Song et al. (2021) [11]	A randomized controlled trial	48	Light	Y	Some concerns	Light intervention is difficult to blind; Some outcomes are subjective.
Mitchell et al. (1997) [12]	A randomized controlled trial	32	Light	Y	Some concerns	Small sample size; No blindness.
Sletten et al. (2017) [13]	A randomized controlled trial	71	Light	Y	Some concerns	No blindness; Subjective outcomes.
Rizza et al. (2022) [14]	A randomized controlled trial	13	Light	Y	High risk	Very small sample size; No blindness.
Griepentrog et al. (2018) [15]	A randomized, crossover clinical trial	43	Light	Y	Some concerns	Crossover design is sensitive to carryover; No blindness.
Nie et al. (2021) [16]	An experimental pilot study	3	Light	Y	High risk	Very small sample size; No randomization; No blindness.
Zanif et al. (2025) [17]	A randomized placebo-controlled trial	40	Melatonin	Y	Low risk	Meets all criteria for low risk.
Hannemann et al. (2024) [18]	A double-blind, randomized, placebo-controlled study	24	Melatonin	Y	Low risk	Meets all criteria for low risk.
Jockovich et al. (2000) [19]	A prospective, randomized, double-blind crossover study	19	Melatonin	N	Some concerns	Small sample size; Crossover design is sensitive to carryover.
James et al. (1998) [20]	A double-blinded, randomized, crossover study	22	Melatonin	N	Some concerns	Crossover design is sensitive to carryover.
Jorgensen et al. (1998) [21]	A double-blind, placebo-controlled crossover trial	18	Melatonin	Y	Some concerns	Small sample size; Crossover design is sensitive to carryover.
Sharkey et al. (2001) [22]	ِA placebo-controlled, double-blind, crossover trial	21	Melatonin	Y	Some concerns	Crossover design is sensitive to carryover.
Sadeghniiat-Haghighi et al. (2008) [23]	A double-blind, placebo-controlled crossover trial	86	Melatonin	Y	Some concerns	Crossover design is sensitive to carryover.
Sharkey and Eastman (2002) [24]	A placebo-controlled simulated night-work study	32	Melatonin	Y	Low risk	Meets all criteria for low risk.
Cavallo et al. (2005) [25]	Double-blind, randomized, placebo-controlled crossover study	28	Melatonin	N	Some concerns	Crossover design is sensitive to carryover.
Smith et al. (2005) [26]	A randomized controlled study	36	Melatonin	N	Low risk	Meets all criteria for low risk.
Wright et al. (1998) [27]	A randomized, placebo-controlled, double-blind, crossover trial	15	Melatonin	N	High risk	Small sample size; Crossover design is sensitive to carryover.
Ning et al. (2020) [28]	Two randomized controlled multicentre trials	450	Melatonin	Y	Low risk	Meets all criteria for low risk.
Gupta et al. (2021) [29]	A randomized controlled trial	39	Diet	Mixed	Some concerns	Blinding is not feasible.
Makowski et al. (2022) [30]	A randomized controlled trial	61	Diet	Mixed	Some concerns	Blinding is not feasible; Partly subjective outcomes.
Leung et al. (2021) [31]	A pilot randomized crossover trial	19	Diet	Mixed	Some concerns	Crossover design is sensitive to carryover.
Sooriyaarachchi et al. (2023) [32]	A randomized controlled clinical trial	8	Diet	Mixed	High risk	Very small sample; Blinding is not feasible.
Silva et al. (2020) [33]	A randomized crossover study	14	Diet	Mixed	High risk	Very small sample; Crossover design is sensitive to carryover; Not blinded; Subjective outcomes.
Oriyama and Yamashita (2021) [34]	A randomized, crossover-controlled, pilot study	15	Diet	Mixed	High risk	Very small sample; Crossover design is sensitive to carryover; Not blinded.
Cunha et al. (2020) [35]	A randomized crossover study	14	Diet	Mixed	Some concerns	Very small sample size; Not blinded.
Al-Naimi et al. (2004) [36]	A randomized crossover study	8	Diet	Mixed	High risk	Very small sample; Crossover design is sensitive to carryover; Not blinded.
Qian et al. (2022) [37]	A randomized controlled experimental study	19	Diet	Mixed	Some concerns	Small sample size; Blinding is not feasible.
de Rijk et al. (2024) [38]	A two-armed randomized crossover trial	51	Diet	Mixed	Some concerns	Crossover design is sensitive to carryover; Not blinded.
Suyoto et al. (2024) [39]	A two-arm randomized crossover trial	53	Diet	Mixed	Some concerns	Crossover design is sensitive to carryover; Blinding is not feasible.
Grant et al. (2017) [40]	A controlled study	11	Diet	Mixed	High risk	Very small sample; Crossover design is sensitive to carryover; Not blinded.
Patterson et al. (2024) [41]	A randomized crossover trial	27	Napping/sleep modification	Mixed	Some concerns	Crossover design is sensitive to carryover; Not blinded.
Oriyama et al. (2014) [42]	A randomized controlled trial	15	Napping/sleep modification	Mixed	High risk	Very small sample size; Not blinded; Subjective outcomes.
Patterson et al. (2023) [43]	A randomized controlled trial	28	Napping/sleep modification	Mixed	Some concerns	Blinding is not feasible.
Patterson et al. (2023) [44]	A randomized crossover trial	26	Napping/sleep modification	Mixed	Some concerns	Crossover design is sensitive to carryover; Not blinded.
Cheng et al. (2022) [45]	A crossover clinical trial	16	Napping/sleep modification	Mixed	Some concerns	Small sample; Crossover design is sensitive to carryover; Not blinded.
Fan et al. (2022) [46]	A randomized controlled trial	105	Napping/sleep modification	Mixed	Some concerns	Not blinded.
Smith-Coggins et al. (2006) [47]	A randomized controlled trial	49	Napping/sleep modification	Mixed	Some concerns	Blinding is not possible; Subjective outcomes.
Oriyama (2024) [48]	A randomized crossover-pilot study	12	Napping/sleep modification	Mixed	High risk	Very small sample size; Crossover design is sensitive to carryover; Not blinded.
Hart et al. (2006) [49]	A controlled trial	11	Modafinil/armodafinil	Y	Some concerns	Small sample size.
Czeisler et al. (2009) [50]	A randomized controlled study	254	Modafinil/armodafinil	Y	Low risk	Low risk of bias across all domains.
Drake et al. (2014) [51]	Randomized, double-blind, crossover study	20	Modafinil/armodafinil	Y	Low risk	Low risk of bias across all domains.
Howard et al. (2014) [52]	A randomized, double-blind, placebo-controlled, crossover study	12	Modafinil/armodafinil	Y	Some concerns	Very small sample size.
Erman et al. (2011) [53]	A multicenter, randomized, double-blind, placebo-controlled, parallel-group clinical trial	383	Modafinil/armodafinil	Y	Low risk	Low risk of bias across all domains.
Walsh et al. (2004) [54]	A double-blind, randomized, parallel groups study	32	Modafinil/armodafinil	Y	Some concerns	Small sample size.
Czeisler et al. (2005) [55]	A randomized double-blind trial	209	Modafinil/armodafinil	Y	Low risk	Low risk of bias across all domains.
Huffmyer et al. (2020) [56]	A randomized controlled trial	26	Caffeine	Y	Some concerns	Small sample size.
Muehlbach and Walsh (1995) [57]	A randomized controlled trial	30	Caffeine	Y	Low risk	Low risk of bias across all domains.
Jay et al. (2006) [58]	A randomized crossover trial	15	Caffeine	Y	High risk	Very small sample size.
Shimada et al. (2011) [59]	A control study	19	Aromatherapy	Y	High risk	Not randomized; Blinding is not possible.
Kim and Hur (2016) [60]	A control study	60	Aromatherapy	Y	High risk	Not randomized; Blinding is not possible; Subjective outcomes.
Nasiri and Boroomand (2021) [61]	A randomized, controlled field trial	80	Aromatherapy	Y	Some concerns	Blinding is not possible; Subjective outcomes.
Hannemann et al. (2020) [62]	A randomized controlled trial	24	Exercise	N	Some concerns	Small sample size; Blinding is not possible.
Lim et al. (2015) [63]	A randomized controlled trial	30	Exercise	Y	Some concerns	Small sample size; Blinding is not possible.
Schäfer et al. (2020) [64]	A randomized controlled trial	64	Exercise	Y	Some concerns	Blinding is not possible.
Miyoshi (2019) [65]	A randomized crossover trial	20	Yoga	Y	High risk	Small sample size; Crossover design is sensitive to carryover; Not blinded; Subjective outcomes.
Wu et al. (2009) [66]	A randomized controlled trial	30	Acupuncture	Y	High risk	Small sample size; Not blinded.
Hwang et al. (2011) [67]	A randomized controlled crossover trial	6	Acupuncture	Y	High risk	Very small sample size; Crossover design is sensitive to carryover; Not blinded.
Walsh et al. (1984) [68]	A controlled trial	10	Triazolam	N	High risk	Very small sample size.
Walsh et al. (1988) [69]	A counterbalanced, crossover trial	18	Triazolam	Y	Some concerns	Crossover design is sensitive to carryover.
Wesensten et al. (2007) [70]	A randomized, double-blind, placebo-controlled, parallel groups trial	48	Ampakine	N	Low risk	Low risk of bias across all domains.
Zeitzer et al. (2020) [71]	A randomized clinical trial	19	Suvorexant	Y	Low risk	Low risk of bias across all domains.
Vallières et al. (2024) [72]	A pilot randomized controlled trial	43	Behavioral therapy	Y	High risk	Not blinded; Subjective outcomes.
Hart et al. (2003) [73]	A within-participant design, residential laboratory study	7	Methamphetamine	Y	Some concerns	Very small sample size.
Kim and Song (2020) [74]	A crossover study	22	Indoor temperature control	Y	Some concerns	Small sample size; Crossover design is sensitive to carryover; Not blinded; Some subjective outcomes.

Results

**Figure 2:**
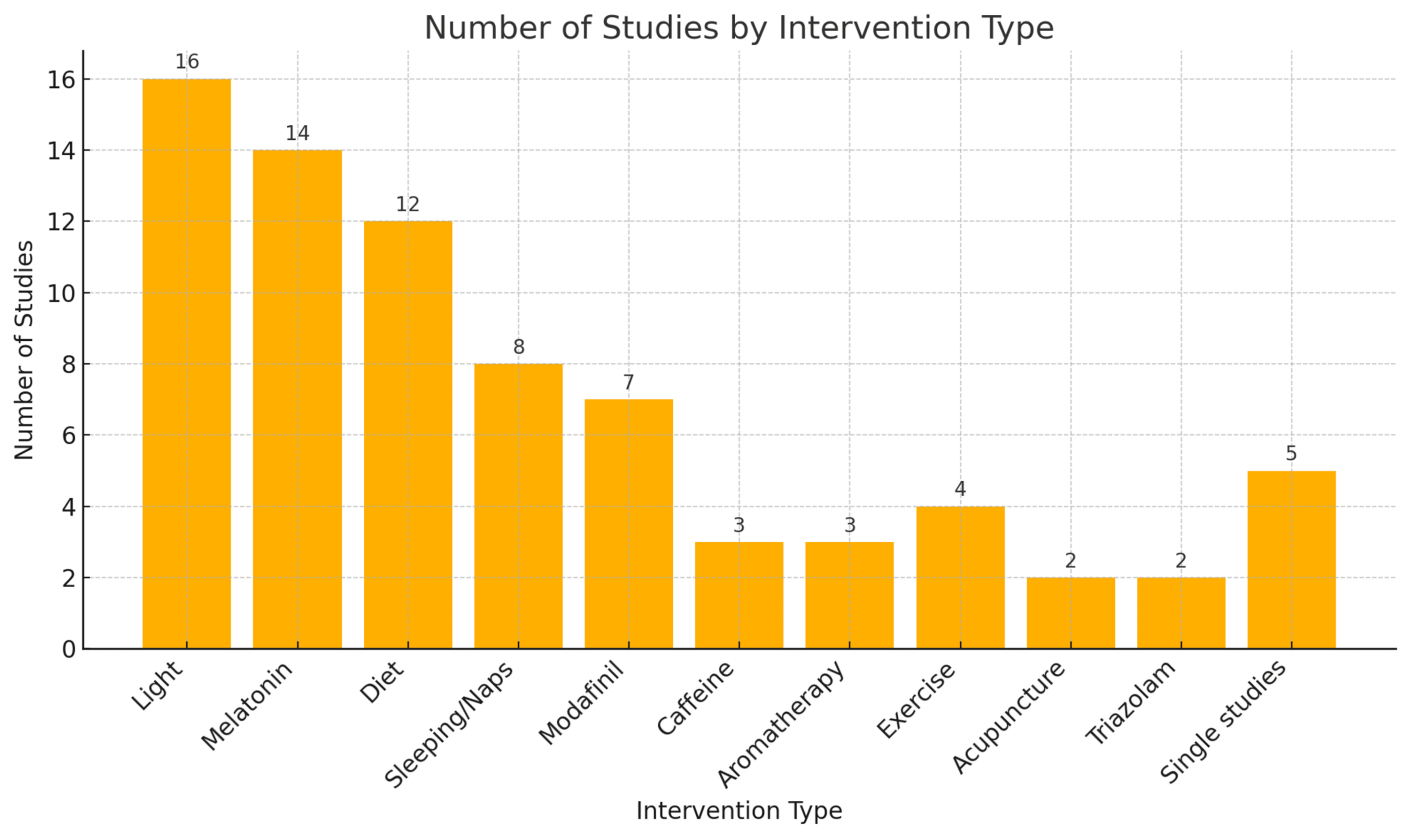
Number of studies by intervention type Image credit: Michael Shakhloul

This systematic review evaluates a range of interventions aimed at reducing the negative effects of night shifts on the health and well-being of doctors and nurses. The studies included in this review (Table 1) examined several types of interventions, grouped into the following categories: light therapy, melatonin, diet, sleep and naps, pharmacological treatments, aromatherapy, physical exercise, acupuncture, and other interventions (Figure 2).

Light Therapy

Among the 16 included studies examining light interventions for night-shift workers [1-16], the majority (n = 12) evaluated the effects of bright light exposure delivered through various protocols, such as continuous, intermittent, or strategically timed pulses [1-3,5-12,15]. These interventions aimed to improve alertness, performance, circadian alignment, and sleep outcomes during or following night shifts.

Notably, 10 of these studies reported positive effects of bright light on key outcomes [2,3,5,6,8-12,15]. These included reductions in subjective sleepiness, enhanced reaction times, and improved circadian adaptation, often measured via melatonin phase shift or sleep-wake consolidation. Timing and spectral composition appeared crucial: bright light applied during the early part of the night shift or just before driving was most effective, particularly when the light was blue-enriched.

In contrast, two trials found no significant benefit of bright light exposure [1,7]. These studies used similar light durations but differed in intensity and timing, suggesting that intervention parameters may significantly influence effectiveness.

Two studies specifically assessed blue-enriched white light compared with standard lighting [11,13]. Both reported improvements in alertness and subjective performance, further supporting the idea that spectral composition, not just intensity, may enhance circadian stimulation and cognitive outcomes.

Finally, two studies tested non-specific light exposure protocols, including ambient environmental light modifications [2,16]. Both reported benefits for sleepiness and psychomotor performance.

Overall, the evidence suggests that light therapy is a promising countermeasure to mitigate the cognitive and physiological challenges associated with night-shift work. However, the heterogeneity in light intensity, duration, spectral composition, and timing complicates cross-study comparison.

Despite encouraging findings, many studies were of short duration, and few assessed sustained adaptation or long-term safety. Additionally, most outcomes were subjective, and blinding was rarely feasible. Future research should focus on standardizing light protocols, assessing individual chronotype responsiveness, and incorporating objective circadian markers in longer-term field settings.

Melatonin

A total of 14 studies assessed the efficacy of exogenous melatonin (n = 13) [2,9,17-27] or the melatonin receptor agonist tasimelteon (n = 1) [28] in mitigating the adverse effects of night-shift work. These interventions aimed to enhance daytime sleep, support circadian realignment, and improve alertness or mood during subsequent night shifts.

Among these, nine studies reported statistically significant benefits following melatonin administration [2,9,17,18,21-24,28]. Improvements were most consistently observed in total sleep time, sleep efficiency, and subjective sleep quality.

The single study evaluating tasimelteon, a dual melatonin receptor agonist, found that it significantly improved both sleep initiation and circadian phase adjustment in shift workers [28]. This suggests potential benefits for agents targeting both MT1 and MT2 receptors with longer half-lives and more consistent bioavailability.

Conversely, five studies reported no statistically significant effects of melatonin on sleep or circadian markers [19,20,25-27]. These null findings may be attributable to low or inconsistent dosing, timing misalignment with circadian phase, or short treatment durations. Additionally, inter-individual differences in melatonin metabolism and chronotype likely contributed to variability in responsiveness.

Notably, few studies used objective circadian markers to verify biological phase shifts. Most relied on subjective sleep ratings or limited actigraphy data, which reduces the certainty of conclusions regarding circadian efficacy.

Collectively, these findings suggest that melatonin-based interventions are promising, particularly for promoting daytime sleep and circadian realignment, but their effects may be dose- and timing-dependent. Personalized approaches that account for chronotype, work schedule, and individual pharmacokinetics may enhance efficacy. Further research should aim to identify optimal dosing regimens, ideal timing relative to shift schedules, and predictors of response to maximize clinical utility.

Dietary Interventions

Twelve studies investigated nutritional interventions for night-shift workers [29-40], exploring the effects of both meal timing and composition on performance, alertness, sleep, and metabolic outcomes. Seven studies focused on the timing and quantity of food intake during the night shift [29,31,34,36-38,40], while four addressed meal composition [30,32,33,35], and one assessed both factors together [39].

Among studies examining meal timing and quantity, findings were mixed. Three trials reported that eating during night shifts, compared to fasting, was associated with improved alertness, reduced fatigue, or enhanced driving performance after shifts [29,31,34]. However, one study found that fasting led to more favorable metabolic outcomes [40]. Other nuanced findings indicated that smaller, more frequent meals might be more beneficial than single large meals. For instance, one study found that three low-glycemic index meals reduced lapses in attention and hunger more effectively than one large meal [38], while another demonstrated that snacking during shifts was associated with better driving performance than consuming a full meal [29].

In terms of dietary composition, three studies reported benefits of low-calorie or low-glycemic index meals over high-calorie or high-glycemic options. These interventions were associated with better glycemic control, reduced hunger, and improved alertness during or after night shifts [30,32,33]. However, two studies found no significant difference between high- and low-calorie meals in terms of performance or metabolic outcomes [35,39], possibly reflecting differences in study design, sample size, or individual variation in metabolic responses.

These findings suggest that nutritional strategies hold promise for mitigating some of the adverse effects of night-shift work. However, results vary depending on the outcome assessed and the intervention approach used. While small, frequent meals or snacks appear to support alertness and reduce hunger, strategic fasting may offer metabolic advantages. Further research is needed to identify personalized nutritional strategies that balance short-term performance needs with long-term health outcomes in shift-working populations.

Sleep and Napping

Our review identified eight studies that evaluated sleep-related interventions [41-48]. Most of these studies focused on the role of strategic napping during or after night shifts, while one addressed the timing of post-shift recovery sleep. Among the six studies investigating naps, consistent evidence supported the benefits of scheduled nap opportunities in improving alertness, cognitive performance, and mood compared to no-nap conditions [41-43,46-48]. Two of these studies further compared short versus long naps, finding that longer naps were associated with superior outcomes [41,44]. 

One study demonstrated that evening sleep following a night shift resulted in better sleep quality and cognitive performance than morning sleep [45]. The study suggests that aligning sleep schedules with individual circadian tendencies can enhance recovery and reduce the cumulative sleep debt associated with consecutive night shifts.

Collectively, these findings indicate that both sleep timing and nap duration are modifiable behavioral factors that can meaningfully improve outcomes for night-shift workers. However, the optimal nap strategy may need to be personalized based on the nature of the shift, individual chronotype, and environmental constraints such as nap opportunities during work. Future studies should explore longer-term outcomes, real-world feasibility, and interactions with other interventions to establish comprehensive fatigue mitigation strategies.

Modafinil/Armodafinil

The reviewed literature included seven studies evaluating the use of modafinil or its R-enantiomer armodafinil [49-55]. Both agents are classified as eugeroics, or wakefulness-promoting drugs, and act primarily as selective dopamine reuptake inhibitors.

All seven studies demonstrated consistent improvements in alertness, vigilance, and cognitive performance during night shifts following administration of either modafinil or armodafinil. Effects were typically measured through validated tools such as the Psychomotor Vigilance Task (PVT), driving simulation tests, and subjective sleepiness scales. In some studies, modafinil or armodafinil was administered 30-60 minutes before the start of the night shift, resulting in statistically significant reductions in attentional lapses, improved reaction time, and enhanced executive functioning, particularly in the early morning hours when fatigue tends to peak.

The consistency of these findings across multiple randomized controlled trials and crossover studies strongly supports the efficacy of these agents in mitigating excessive sleepiness associated with night work.

In summary, the available evidence suggests that modafinil and armodafinil are reliable and well-tolerated pharmacological options for promoting wakefulness and reducing performance deficits during night shifts. While highly effective in the short term, their use should be guided by clinical indications, and caution is advised regarding long-term use, potential masking of underlying sleep deficits, and regulatory restrictions in occupational settings.

Caffeine

Three studies included in this review assessed the impact of caffeine on alertness and performance in night-shift workers [56-58]. All three studies consistently demonstrated that caffeine significantly improved alertness, vigilance, and psychomotor performance during or after night shifts.

The reviewed studies employed various forms of caffeine administration - including caffeinated beverages and energy drinks - with different dosages, and timing varied from early in the shift to just before post-shift driving tasks. Nevertheless, beneficial effects were observed across all conditions, suggesting a robust wakefulness-promoting effect that is relatively insensitive to modest variations in timing or delivery.

In summary, caffeine appears to be an effective and practical intervention for combating night shift-related sleepiness. Its consistent performance across studies, regardless of timing and dosage, reinforces its utility as a frontline option for managing fatigue in night-shift workers. 

Aromatherapy

Three studies in this review explored aromatherapy - the therapeutic use of plant-derived essential oils and aromatic compounds - as a non-pharmacological intervention for night-shift workers [59-61]. Despite variations in study design, essential oil type, and application method, all three studies reported positive outcomes, including improvements in subjective alertness, reduced sleepiness, and enhanced sleep quality following night shifts. These findings suggest that olfactory stimulation through aromatherapy may offer a simple, low-risk approach to alleviating some of the adverse effects associated with night work.

While these early findings are encouraging, several limitations warrant caution. The sample sizes were modest, and aromatherapy trials are inherently difficult to blind, increasing the risk of placebo effects. Moreover, the heterogeneity of essential oil types, concentrations, and delivery methods makes it difficult to establish standardized protocols or directly compare efficacy. Studies incorporated objective measures, but the duration of interventions was short, limiting conclusions about sustained benefits.

In summary, aromatherapy appears to be a promising adjunctive strategy for supporting alertness and sleep recovery in night-shift workers. Given its non-invasive nature and ease of implementation, it may serve as a complementary approach to other behavioral or pharmacological interventions. However, larger, controlled trials with objective outcome measures are needed to confirm its efficacy and determine optimal formulations and timing for use in shift work populations.

Physical Exercise

Four studies examined the effects of exercise and physical activity interventions on outcomes relevant to night-shift workers, with mixed but potentially promising results [62-65]. These studies explored a range of activity types and assessed outcomes including stress, circadian rhythms, cardiovascular markers, and physical work capacity.

The most consistently positive finding was observed in the study evaluating restorative yoga, which demonstrated significant reductions in occupational stress among female nurses working night shifts [65]. Participants in this trial reported improved emotional well-being following guided yoga sessions and at-home practice, highlighting the potential of low-intensity, parasympathetic-activating movement to counteract the psychological strain of night work.

Among studies examining conventional physical exercise, two reported favorable effects. One trial found that intermittent aerobic training over a 10-week period improved biomarkers of cardiovascular risk, such as lipid profiles and inflammatory markers, in night shift workers [63]. Another study showed that high-intensity interval training (HIIT) before night shifts improved arterial stiffness and physical work capacity, both of which are predictive of long-term cardiovascular outcomes [64]. These results suggest that exercise interventions may offer protective physiological effects, potentially offsetting some of the cardiometabolic risks associated with shift work.

In contrast, one study reported no significant improvements in circadian rhythms or glucose tolerance following a series of low-intensity exercise sessions conducted before night shifts [62]. This discrepancy may stem from variations in exercise type, duration, and intensity, as well as differences in outcome measures and participant characteristics across studies. None of the trials directly compared different exercise modalities or examined long-term adherence and feasibility in real-world occupational settings.

Taken together, the preliminary evidence suggests that physical activity interventions, particularly mind-body exercises like restorative yoga, may offer benefits for stress reduction and cardiovascular health in night-shift workers. However, findings for traditional exercise regimens are inconclusive, and further research is required to determine the optimal type, timing, and intensity of physical activity to support this population. Future studies should prioritize standardized protocols, larger sample sizes, and longitudinal designs to clarify the role of exercise in mitigating the physiological and psychological demands of night work.

Acupuncture

Two studies investigated acupuncture as an intervention for night shift workers [66,67], with both demonstrating significant effects on autonomic nervous system regulation. The studies consistently reported that acupuncture treatment resulted in decreased sympathetic activity coupled with increased parasympathetic tone, suggesting its potential to correct the autonomic imbalance commonly associated with circadian disruption in shift work. These neurophysiological changes may underlie acupuncture's proposed benefits for shift workers, as autonomic dysregulation has been implicated in various shift work-related health consequences. The concordant findings across both studies, despite potential differences in acupuncture protocols, provide preliminary evidence for this traditional Chinese medicine technique as a viable complementary approach for managing the physiological stressors of night work. However, the small number of available studies indicates the need for more extensive research to validate these effects and establish optimal treatment parameters.

Triazolam

The review identified two studies examining triazolam, a short-acting benzodiazepine typically prescribed for acute insomnia and circadian rhythm sleep disorders, in the context of shift work adaptation [68,69]. The findings presented a notable discrepancy: while one study reported no significant improvement in daytime sleep quality among night shift workers [68], the other demonstrated measurable benefits for daytime sleep duration and quality [69]. These mixed results underscore the need for additional controlled studies to clarify triazolam's role in shift work disorder management and to identify the specific conditions under which it may be beneficial, particularly given concerns about potential tolerance development and rebound insomnia associated with benzodiazepine use.

Single Studies

Our review identified several interventions examined by single studies, each demonstrating varying degrees of potential for managing shift work challenges. The ampakine compound CX717 showed no significant effect in reversing night shift-related performance and alertness deficits [70], while suvorexant, an orexin receptor antagonist, demonstrated efficacy in improving daytime sleep duration [71]. Behavioral therapy specifically designed for shift work disorder emerged as a promising non-pharmacological approach, showing benefits for both sleep quality and mental health in healthcare night workers [72]. Pharmacologically, low-to-moderate dose methamphetamine was found to attenuate shift change-related performance and mood disruptions [73], though this approach warrants cautious consideration due to abuse potential. Interestingly, an environmental intervention study suggested that maintaining an indoor temperature of 23°C may positively influence night shift adaptation [74]. These isolated findings highlight diverse potential avenues for shift work management while underscoring the need for replication studies to establish their efficacy and safety profiles more definitively.

## Conclusions

This review identifies several promising strategies to mitigate the adverse effects of night-shift work. These interventions show potential to improve alertness, sleep, and circadian alignment, though their effectiveness varies based on timing, individual factors, and occupational context. Limitations across studies, including heterogeneous designs, short durations, and inconsistent outcomes, restrict definitive conclusions.

Pharmacological approaches may be effective but require careful consideration of side effects and long-term safety. Overall, a personalized, multimodal approach combining behavioral strategies with selective pharmacologic support appears most promising. Future research should prioritize standardized protocols, objective outcomes, and long-term feasibility to inform evidence-based, sustainable solutions for night-shift workers.
